# Acute Effects of *Hom Pathum* and *Tubtim Chumphae* Rice Jellies on Glycemic Response, Endurance Performance, and Oxidative Stress in Healthy Adults: A Randomized Crossover Study

**DOI:** 10.3390/foods15122122

**Published:** 2026-06-12

**Authors:** Orachorn Boonla, Uraiporn Booranasuksakul, Pongrung Chancharoen, Thapanee Roengrit, Promtpong Anuchitchanchai, Piyapong Prasertsri

**Affiliations:** 1 Faculty of Allied Health Sciences, Burapha University, Chonburi 20131, Thailand; orachorn@go.buu.ac.th (O.B.); uraiporn@go.buu.ac.th (U.B.); pongrung@go.buu.ac.th (P.C.); 2 Institute of Medicine, Suranaree University of Technology, Nakhon Ratchasima 30000, Thailand; thapanee.ro@sut.ac.th; 3 Department of Orthopedics, Faculty of Medicine, Burapha University, Chonburi 20131, Thailand; promtpong@yahoo.co.th

**Keywords:** Thai rice, glycemic response, endurance performance, oxidative stress, anthocyanins

## Abstract

Thai rice varieties, including *Hom Pathum* (*Pathumthani* fragrant rice) and *Tubtim Chumphae* rice, contain bioactive compounds with potential antioxidant properties. In this study, their acute effects on glycemic response, cardiac autonomic function, endurance performance, and oxidative stress were investigated in healthy adults. In a randomized crossover design, two independent cohorts of healthy adults were enrolled. A total of 50 participants completed two separate experiments (*n* = 25 per experiment), in which they consumed 140 g of control jelly, *Hom Pathum* rice jelly, and *Tubtim Chumphae* rice jelly in a randomized order on separate occasions. In Experiment 1, blood glucose (BG) was measured at baseline and every 30 min for 120 min, while insulin concentrations were assessed at baseline and after 120 min. In Experiment 2, participants performed treadmill exercise at 60% VO_2_peak to volitional exhaustion, and exercise-induced oxidative stress was evaluated following exercise. Postprandial BG responses differed significantly among interventions. At 30 min, BG concentrations were lower following *Hom Pathum* and *Tubtim Chumphae* rice jellies compared with the control jelly (*p* < 0.001 and *p* = 0.002, respectively), and these reductions were maintained between 60 and 120 min, with *Tubtim Chumphae* rice generally demonstrating greater glycemic attenuation (*p* < 0.05). The BG area under the curve was significantly lower following both rice jellies than following the control jelly (*p* ≤ 0.005). No significant differences were observed in insulin concentrations, HOMA indices, heart rate variability, or blood pressure among interventions. Both rice jellies improved endurance performance compared with the control condition (*p* < 0.05), whereas post-exercise malondialdehyde concentrations were significantly reduced only following *Tubtim Chumphae* rice consumption (*p* = 0.049). These findings suggest that acute consumption of Thai rice-based jellies, particularly *Tubtim Chumphae* rice jelly, may attenuate postprandial glycemic responses and enhance endurance performance, with *Tubtim Chumphae* rice additionally demonstrating potential to reduce exercise-induced oxidative stress. However, these findings reflect short-term physiological responses in healthy adults and should be interpreted cautiously pending further mechanistic and long-term investigations.

## 1. Introduction

Rice (*Oryza sativa* L.) is a staple food consumed by a substantial proportion of the global population, particularly in Asia, where it contributes considerably to daily energy intake. Beyond its role as a major carbohydrate source, certain rice varieties contain bioactive compounds that may exert physiological and health-promoting effects [1]. Thai rice cultivars, including *Hom Pathum* (*Pathumthani* fragrant rice) and *Tubtim Chumphae* rice, contain diverse phytochemicals such as phenolic compounds and flavonoids, while pigmented varieties additionally provide anthocyanins with potent antioxidant activity [2]. These bioactive constituents have attracted growing interest because of their potential roles in metabolic regulation and disease prevention [3].

Postprandial glycemic response is an important determinant of metabolic health, as exaggerated postprandial glucose excursions have been associated with increased risks of insulin resistance [4], type 2 diabetes [5], and cardiovascular disease [6]. Consequently, dietary approaches aimed at attenuating postprandial glycemia have become an important focus of nutritional and functional-food research. In addition to carbohydrate quantity, emerging evidence suggests that carbohydrate quality and associated phytochemicals may influence broader physiological outcomes, including cardiovascular regulation [7], exercise performance [8], and oxidative stress [9], particularly during endurance exercise [10]. Exercise-induced oxidative stress has been implicated in fatigue development and post-exercise recovery, and antioxidant-rich foods may help attenuate these responses through redox homeostasis modulation [11,12].

Pigmented rice varieties have increasingly been recognized as functional foods because they provide not only starch and essential nutrients but also a wide range of bioactive compounds, including phenolics, flavonoids, anthocyanins, tocopherols, and γ-oryzanol [13]. Many of these compounds exhibit antioxidant and anti-inflammatory properties and may contribute to the regulation of glucose metabolism, oxidative balance, and cardiovascular function [14]. Anthocyanins, responsible for the characteristic red, purple, and black pigmentation of rice grains, possess strong free radical scavenging capacity and have been implicated in cellular protection and metabolic regulation [15]. Notably, the composition and concentration of these phytochemicals vary considerably among rice cultivars and may be further modified using processing and preparation methods, potentially contributing to differences in physiological responses following consumption.

Growing interest in functional food research has prompted investigations into whether phytochemical-rich carbohydrate sources influence physiological outcomes beyond caloric provision alone. Previous studies suggest that bioactive-rich rice varieties may modulate postprandial glycemic responses through several mechanisms, including altered starch digestibility, delayed glucose absorption, inhibition of carbohydrate-digesting enzymes, and antioxidant-mediated regulation of metabolic pathways [16,17,18]. Furthermore, antioxidant-rich foods may attenuate exercise-induced oxidative stress and thereby support endurance performance and physiological recovery. However, despite increasing evidence regarding the bioactive potential of pigmented rice, human studies examining the acute physiological effects of Thai rice varieties remain limited. In particular, studies that evaluate both metabolic and functional outcomes and directly compare pigmented with commonly consumed non-pigmented rice cultivars are scarce. Consequently, the comparative effects of Thai rice varieties on glycemic regulation, cardiac autonomic function, endurance performance, and oxidative stress remain insufficiently characterized.

Therefore, this study aimed to investigate the acute effects of *Hom Pathum* and *Tubtim Chumphae* rice consumption on postprandial glycemic response, cardiac autonomic function, endurance performance, and oxidative stress in healthy adults using a randomized crossover design.

## 2. Materials and Methods

### 2.1. Study Design and Sample Size

This study was designed as a randomized, single-blind, placebo-controlled crossover trial comprising two independent experiments conducted in separate cohorts of healthy adults. Experiment 1 (*n* = 25) investigated the acute effects of *Hom Pathum* rice jelly and *Tubtim Chumphae* rice jelly on blood glucose (BG), insulin concentrations, Homeostatic Model Assessment (HOMA) indices, and cardiac autonomic function. Experiment 2 (*n* = 25) evaluated the effects of the same interventions on endurance performance and exercise-induced oxidative stress. Because each experiment enrolled a distinct cohort of participants and addressed different primary outcomes, the datasets were analyzed independently. Consequently, the total study population comprised 50 participants.

The sample size was determined a priori using a standard approach for crossover studies. The estimation was based on data from a previous randomized crossover study evaluating the acute effects of passion fruit juice supplementation on postprandial BG responses in healthy individuals [19], which reported a mean between-condition difference of 16.4 ± 9.0 mg/dL. Assuming a two-sided type I error (α) of 0.05 and a type II error (β) of 0.20, corresponding to 80% statistical power, the minimum required sample size was estimated to be 22 participants. To account for an anticipated dropout rate of approximately 10%, the target sample size was increased to 25 participants.

Although the sample size estimation was derived from a fruit-based carbohydrate intervention, this reference was considered methodologically appropriate because it employed a comparable randomized crossover design and assessed a similar acute postprandial glycemic outcome. Consequently, the selected sample size was considered suitable for evaluating intervention-related differences in this study.

Post hoc analyses were additionally conducted to evaluate effect sizes and achieved statistical power for the primary outcomes. For the BG area under the curve (AUC), the intervention effect demonstrated a large effect size (partial eta squared, η^2^_p_ ≈ 0.22) with high achieved power (approximately 0.99). Similarly, endurance performance exhibited a large effect size (η^2^_p_ ≈ 0.22) and high achieved power (approximately 0.99). These findings support the adequacy of the study sample size and indicate sufficient statistical sensitivity to detect meaningful intervention effects for the primary outcomes.

### 2.2. Ethical Considerations

This study was conducted in accordance with the ethical principles of the Declaration of Helsinki and was approved by the Human Research Ethics Committee of Burapha University (Approval No. IRB1-047/2567; approved on 2 May 2024). The study was retrospectively registered at ClinicalTrials.gov (Identifier: NCT06475222; registered on 20 June 2024). Although registration occurred after study initiation, the study protocol, eligibility criteria, outcome measures, and statistical analysis plan were prospectively established and remained unchanged throughout the study period. Written informed consent was obtained from all participants prior to enrollment and participation in this study.

### 2.3. Participants and Screening

Participants were screened using a structured health questionnaire that included a COVID-19 symptom checklist, demographic information, medical history, and information regarding dietary supplement use, food allergies, smoking status, alcohol consumption, and exercise habits. In addition, anthropometric and physiological assessments were performed, including body composition, blood pressure, heart rate, and body temperature measurements.

The inclusion criteria were as follows: (1) male or female participants aged 18–35 years; (2) apparently healthy without diagnosed underlying diseases; and (3) willingness to consume jelly prepared from *Hom Pathum* and *Tubtim Chumphae* rice. The exclusion criteria included the following: (1) history of allergy to *Hom Pathum* rice, *Tubtim Chumphae* rice, or carrageenan; (2) body mass index (BMI) ≥ 25 kg/m^2^, according to World Health Organization criteria; (3) regular smoking or alcohol consumption; (4) regular participation in structured exercise or training programs; (5) regular use of dietary supplements; (6) musculoskeletal or other medical conditions that could interfere with treadmill exercise performance; and (7) signs or symptoms suggestive of infection or inflammation, including fever.

To enhance participant safety, all eligible participants additionally received a small test dose of *Hom Pathum* rice jelly and *Tubtim Chumphae* rice jelly (approximately 50 mg each) to assess potential immediate allergic reactions or adverse symptoms following acute ingestion. No immediate adverse reactions were observed.

### 2.4. Preparation of Jellies

The *Hom Pathum* and *Tubtim Chumphae* rice used in this study were obtained from the Ban Wat Khu Kasem Samakkee Rice Processing Community Enterprise, Khlong Udom Chonlachon Subdistrict, Mueang Chachoengsao District, Chachoengsao Province, Thailand, a region recognized for high-quality rice production.

The ingredients used for jelly preparation consisted of *Hom Pathum* or *Tubtim Chumphae* rice, glucose syrup, and carrageenan powder. Rice grains were thoroughly washed and boiled in water prior to rice milk extraction. The boiling duration was 15 min for *Hom Pathum* rice and 30 min for *Tubtim Chumphae* rice. After cooking, rice milk was obtained by filtering the cooked rice through muslin cloth. The extracted rice milk was mixed with glucose syrup, followed by the addition of carrageenan powder to form the jelly mixture. The mixture was then poured into prepared containers, allowed to cool at room temperature (22–28 °C) for approximately 30 min, and subsequently stored under refrigerated conditions (2–4 °C) until use.

The control jelly was prepared using an identical carrageenan-based formulation and standardized preparation procedure, with glucose syrup substituted for rice milk to provide comparable energy content while excluding rice-derived constituents. The control jelly provided 40 kcal/100 g, which was comparable to the energy content of the *Hom Pathum* and *Tubtim Chumphae* rice jellies (39.9 and 39.6 kcal/100 g, respectively). The nutritional compositions of the rice jellies were subsequently analyzed and are presented in Table 1.

Although the interventions were formulated to achieve comparable caloric values and broadly similar sugar composition, the control jelly differed from the rice-based jellies in carbohydrate source and lacked rice-derived starch and phytochemical constituents.

The jelly preparation protocol was adapted from a Thai publication on fruit jelly development and our previous study [20], with modifications to accommodate the physicochemical characteristics of rice. To ensure formulation standardization and minimize batch-to-batch variability, all rice materials were obtained from the same harvest, and all jelly was prepared by a single experienced food preparer using standardized procedures.

### 2.5. Protocols of Experiment 1

Prior to each experimental session, participants reported to the laboratory in the morning following an overnight fast of 8–10 h, during which only water was permitted. Participants were additionally instructed to refrain from alcohol consumption, strenuous physical activity, and unusual dietary intake for 24 h before testing.

**Figure 1:**
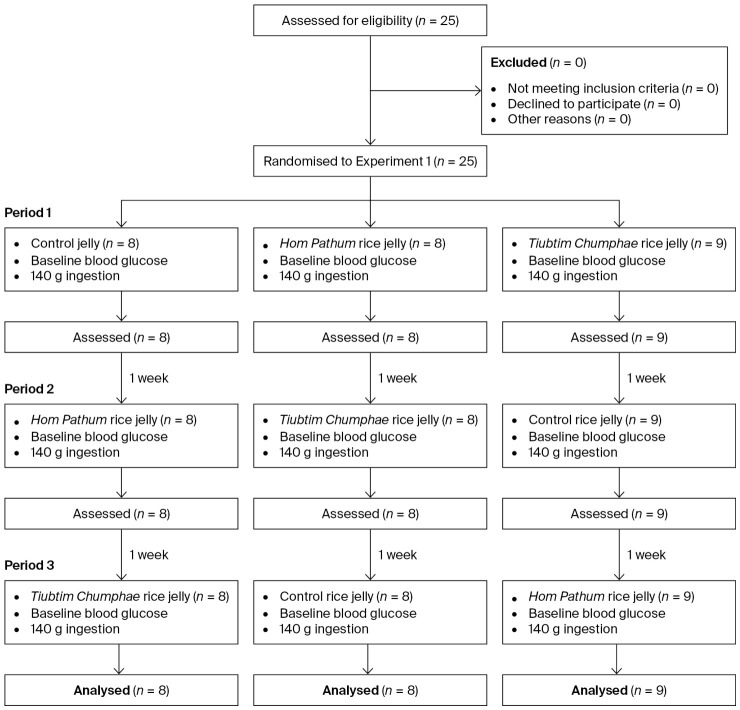
CONSORT flow diagram of participant recruitment, randomization, crossover allocation, and analysis for Experiment 1 (glycemic and cardiovascular assessments). Twenty-five healthy adults consumed control jelly, *Hom Pathum* rice jelly, and *Tubtim Chumphae* rice jelly in randomized order with a 1-week washout between conditions. Blood glucose, insulin, HOMA indices, and cardiac autonomic responses were assessed following each intervention.

As illustrated in Figure 1, participants consumed 140 g of either the control jelly, *Hom Pathum* rice jelly, or *Tubtim Chumphae* rice jelly in a randomized crossover sequence. Following each intervention, BG, insulin concentrations, HOMA indices, and cardiac autonomic responses were evaluated over a 120 min postprandial period.

BG concentrations were measured at baseline (0 min) and at 30, 60, 90, and 120 min following jelly ingestion, and the AUC for BG was subsequently calculated. Insulin concentrations were assessed at baseline and 120 min, and HOMA indices were derived accordingly. Cardiac autonomic nervous system activity was evaluated using heart rate variability (HRV), which was recorded for 10 min before jelly consumption (baseline) and continuously monitored throughout the 120 min post-ingestion period.

A 1-week washout period was implemented between intervention conditions to minimize potential carryover effects. Following each washout period, participants crossed over to the subsequent intervention and completed the same experimental procedures.

### 2.6. Protocols of Experiment 2

**Figure 2:**
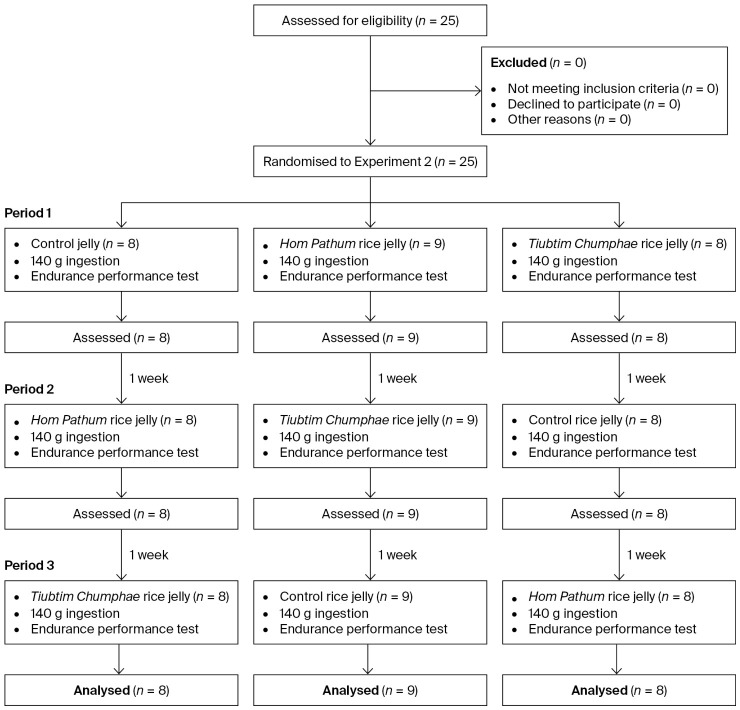
CONSORT flow diagram of participant recruitment, randomization, crossover allocation, and analysis for Experiment 2 (endurance and oxidative stress assessments). Twenty-five healthy adults consumed control jelly, *Hom Pathum* rice jelly, and *Tubtim Chumphae* rice jelly in randomized order with a 1-week washout between conditions. Endurance performance and oxidative stress biomarkers were evaluated following each intervention.

As illustrated in Figure 2, participants consumed 140 g of either the control jelly, *Hom Pathum* rice jelly, or *Tubtim Chumphae* rice jelly according to a randomized crossover sequence. Thirty minutes following jelly ingestion, endurance performance was evaluated using a treadmill running test performed at 60% of each participant’s peak oxygen consumption (VO_2_peak) until volitional exhaustion.

HR, dyspnea, and leg fatigue scores were recorded at the point of exhaustion. Immediately following completion of the endurance test, venous blood samples were collected for the determination of oxidative stress biomarkers.

Consistent with Experiment 1, a 1-week washout period was implemented between intervention conditions to minimize potential carryover effects. Following each washout period, participants crossed over to the subsequent intervention and completed the same experimental procedures.

All participants completed both Experiments 1 and 2 within the same study period under comparable environmental conditions, including controlled room temperature and relative humidity. Throughout the study, participants were instructed to maintain their habitual lifestyle behaviors, particularly with respect to dietary intake and physical activity, to minimize potential confounding influences on study outcomes.

### 2.7. Primary and Secondary Outcomes and Measurements

#### 2.7.1. Glycemic Control and HOMA Indices Measurements

In Experiment 1, BG concentrations (mg/dL) were measured at baseline (0 min) and at 30, 60, 90, and 120 min post-ingestion using the Accu-Chek^®^ Guide BG monitoring system (Roche Diabetes Care Inc., Indianapolis, IN, USA), following procedures described in our previous study [19]. The AUC for BG was calculated over the 120 min period.

Approximately 3 mL of venous blood was collected into clot activator tubes for the analysis of insulin concentrations (µU/mL). Insulin levels were determined using the ARCHITECT Insulin assay (Abbott Laboratories, Abbott Park, IL, USA) and analyzed by RIA Laboratory Co., Ltd. (Chonburi, Thailand).

HOMA indices were calculated using standard equations [21,22,23].

HOMA-IR was calculated as follows: fasting glucose (mg/dL) × fasting insulin (µU/mL)/405.

HOMA-B was calculated as follows: 20 × fasting insulin (µU/mL)/[fasting glucose (mg/dL) − 63].

HOMA-%S was calculated as follows: (1/HOMA-IR) × 100.

QUICKI was calculated as follows: 1/[log(fasting insulin (µU/mL)) + log(fasting glucose (mg/dL))].

#### 2.7.2. Cardiac Autonomic Nervous System Measurement

Cardiac autonomic nervous system activity was assessed using HRV analysis. Electrocardiographic signals were recorded using a lead II configuration (PowerLab 4/30, AD Instruments, Bella Vista, NSW, Australia) and analyzed with the HRV module of LabChart^®^ Pro software Version 7 (AD Instruments, Bella Vista, NSW, Australia).

HRV indices included time-domain parameters, namely the standard deviation of normal-to-normal intervals (SDNN) and the root mean square of successive R–R interval differences (RMSSD), as well as frequency-domain parameters, including low-frequency (LF) power (0.04–0.15 Hz), high-frequency (HF) power (0.15–0.40 Hz), and the LF/HF ratio. LF power is generally considered to reflect predominantly sympathetic modulation, whereas HF power is associated with parasympathetic (vagal) activity. The LF/HF ratio is commonly interpreted as an index of sympathovagal balance [24].

#### 2.7.3. Blood Pressure Measurement

Blood pressure (BP) and HR were measured under resting conditions before and after jelly ingestion using an automated digital BP monitor (HEM-7121, Omron Healthcare Co., Ltd., Kyoto, Japan). The cuff was placed on the upper arm, with the lower edge positioned approximately 2–3 cm above the antecubital crease.

Two consecutive measurements were obtained at 1 min intervals, and the average of the two readings was used to determine systolic BP (SBP) and diastolic BP (DBP). Pulse pressure (PP), mean arterial pressure (MAP), and rate–pressure product (RPP) were subsequently calculated using standard equations: PP = SBP − DBP; MAP = DBP + PP/3; RPP = HR × SBP.

#### 2.7.4. VO_2_peak and Endurance Performance Assessments

Participants underwent a submaximal exercise test using the Bruce protocol to estimate VO_2_peak and determine the workload corresponding to 60% VO_2_peak. To assess VO_2_peak, participants completed a continuous incremental exercise test to voluntary exhaustion on a calibrated treadmill ergometer (Lode Valiant; Lode BV, Groningen, The Netherlands) using MetaSoft Studio Version 5.13.0 SR2 (MetaMax 3B, Cortex, Leipzig, Germany).

Briefly, the test began at a speed of 2.7 km·h^−1^ with a 10% incline for 3 min. Workload (speed and grade) was subsequently increased every 3 min in a stepwise manner until volitional exhaustion was reached [25]. Exhaustion was defined as the onset of maximal dyspnea and fatigue or the inability to maintain the required exercise intensity.

At a separate visit, conducted 1 week after the VO_2_peak assessment, endurance performance was evaluated as time to exhaustion during treadmill running exercise at 60% VO_2_peak. HR, dyspnea, and leg fatigue were recorded at the point of exhaustion. Perceptual responses were assessed using the Borg rating of perceived exertion (RPE; 6–20 scale) for leg fatigue and the modified Borg rating of perceived dyspnea (RPD; 0–10 scale) for breathlessness.

#### 2.7.5. Oxidative Stress Biomarker Assay

Approximately 1 mL of venous blood was collected into ethylenediaminetetraacetic acid (EDTA) tubes to assess lipid peroxidation as an indicator of oxidative stress. Plasma malondialdehyde (MDA) levels were determined using the thiobarbituric acid reactive substances (TBARS) assay. Briefly, 150 μL of plasma was mixed with trichloroacetic acid (10%), EDTA (5 mM), sodium dodecyl sulfate (8%), and butylated hydroxytoluene (0.5 μg/mL), followed by the addition of TBA (0.6%). The mixture was incubated in boiling water for 30 min. After incubation, samples were centrifuged at 10,000 rpm for 5 min at room temperature (Eppendorf Centrifuge 5810R, Hamburg, Germany), and the absorbance of the supernatant was measured at 532 nm using a UV/Vis Basic BioSpectrometer (Eppendorf, Hamburg, Germany). A standard curve was generated using 1,1,3,3-tetraethoxypropane (0.1–10 μmol/L) to quantify MDA levels [26].

### 2.8. Statistical Analyses

Data were assessed for normality using the Shapiro–Wilk test, homogeneity of variance using Levene’s test, and sphericity using Mauchly’s test.

In Experiment 1, changes in BG concentrations and HRV over time (0, 30, 60, 90, and 120 min) within and between ingestion conditions (control, *Hom Pathum* rice jelly, and *Tubtim Chumphae* rice jelly) were analyzed using repeated-measures analysis of covariance (ANCOVA), with the baseline value (0 min) included as a covariate. In addition, differences in insulin concentrations and HOMA indices among interventions were analyzed using one-way analysis of variance (ANOVA) followed by Tukey’s post hoc test. Pairwise comparisons were further examined using paired *t*-tests.

In Experiment 2, differences in outcome variables (endurance performance and oxidative stress biomarkers) among interventions were analyzed using one-way ANOVA with Tukey’s post hoc test.

All statistical analyses were conducted using IBM SPSS Statistics for Windows (Version 25, IBM Corp., Armonk, NY, USA). Data are presented as mean ± standard deviation (SD), and statistical significance was set at *p* < 0.05.

## 3. Results

### 3.1. Experiment 1

Twenty-five participants were enrolled, and they all (100%) completed the study. Therefore, data from all participants were included in the final analysis. The sex distribution was comparable (*p* = 0.841), with 12 (48%) male and 13 (52%) female participants.

#### 3.1.1. Baseline Physical and Physiological Characteristics of Participants in Experiment 1

The baseline physical and physiological characteristics of the participants are presented in Table 2. Physical characteristics included age, body weight, BMI, body fat percentage, subcutaneous fat percentage, muscle percentage, visceral fat level, and basal metabolic rate. Physiological characteristics included HR, SBP, and DBP.

#### 3.1.2. Blood Glucose Control Response

Baseline insulin, HOMA-B, HOMA-%S, and QUICKI were comparable among the three interventions, whereas baseline BG and HOMA-IR differed significantly (*p* < 0.05). Therefore, time-dependent responses were analyzed using two-way ANCOVA with baseline adjustment.

**Figure 3:**
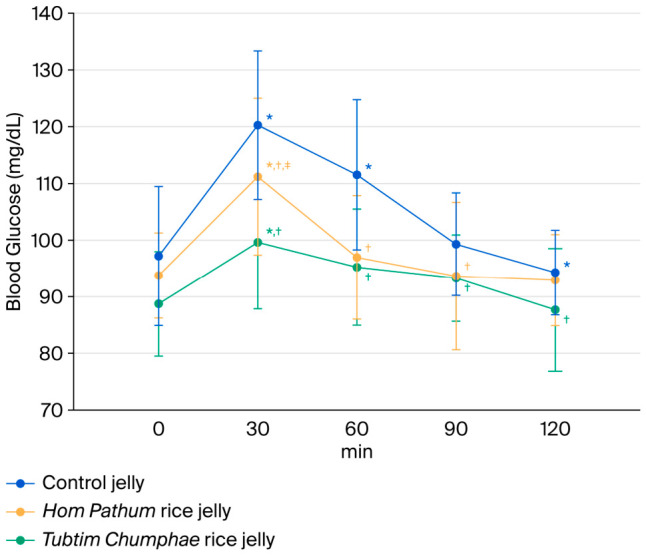
Blood glucose concentrations measured at baseline and 30, 60, 90, and 120 min following ingestion of control jelly, *Hom Pathum* rice jelly, or *Tubtim Chumphae* rice jelly. Data are presented as mean ± SD (*n* = 25). Statistical comparisons were performed using repeated-measures ANCOVA with baseline adjustment. * *p* < 0.05 vs. baseline; ^†^ *p* < 0.05 vs. control jelly; ^‡^ *p* < 0.05 vs. *Tubtim Chumphae* rice jelly.

No significant between-intervention differences were observed for insulin, HOMA-IR, HOMA-B, HOMA-%S, or QUICKI (all *p* > 0.05). In contrast, BG differed significantly among interventions. At 30 min, BG was lower following *Hom Pathum* and *Tubtim Chumphae* rice jellies compared with the control (*p* < 0.001 and *p* = 0.002, respectively) and was higher in the *Hom Pathum* than in the *Tubtim Chumphae* intervention (*p* = 0.016). At 60 min, BG remained lower in both rice jelly interventions than in the control (both *p* < 0.001). Similarly, at 90 min, BG was reduced in the *Hom Pathum* and *Tubtim Chumphae* interventions compared with the control (*p* = 0.011 and *p* = 0.039, respectively). By 120 min, BG remained significantly lower only in the *Tubtim Chumphae* intervention compared with the control (*p* = 0.043) (Figure 3; Table 3).

**Figure 4:**
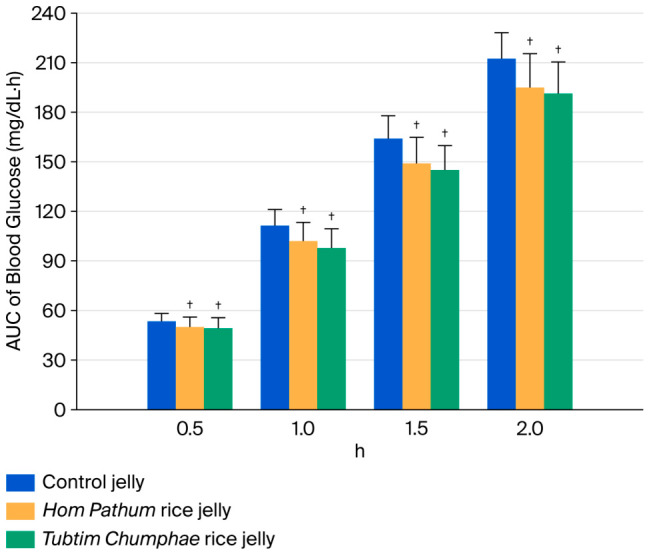
Area under the blood glucose curve (AUC) during the 2 h postprandial period following ingestion of control jelly, *Hom Pathum* rice jelly, or *Tubtim Chumphae* rice jelly. Data are presented as mean ± SD (*n* = 25). Statistical analyses were performed using repeated-measures ANCOVA with baseline adjustment. ^†^ *p* < 0.05 vs. control jelly.

BG AUC analysis confirmed these findings. Control jelly showed consistently higher AUC values across all time intervals (0–0.5, 0–1.0, 0–1.5, and 0–2.0 h) compared with both the *Hom Pathum* rice (*p* = 0.005 to *p* < 0.001) and *Tubtim Chumphae* rice interventions (*p* = 0.019 to *p* = 0.001) (Figure 4; Table 3).

Within-group analyses indicated that BG peaked at 30 min in all interventions. In the control intervention, BG at 30 and 60 min was higher than at baseline (*p* < 0.001 and *p* = 0.013), and 30 min values were higher than at all other time points (all *p* < 0.001). In the *Hom Pathum* intervention, BG at 30 and 60 min exceeded baseline (both *p* < 0.001), with the 30 min values being higher than those at 90 and 120 min (both *p* < 0.001). In the *Tubtim Chumphae* intervention, BG at 30 min was higher than that at baseline and at 60–120 min (*p* = 0.008–0.001).

At 120 min, insulin and HOMA-IR decreased significantly, and HOMA-B increased significantly from baseline in the *Hom Pathum* (*p* = 0.013, *p* = 0.016, and *p* = 0.026, respectively) and *Tubtim Chumphae* interventions (*p* = 0.006, *p* = 0.009, and *p* = 0.030, respectively), whereas HOMA-%S increased in the *Tubtim Chumphae* intervention (*p* = 0.038). In the control intervention, only insulin decreased significantly at 120 min (*p* = 0.032).

#### 3.1.3. Heart Rate Variability Response

Baseline HRV indices did not differ among interventions (all *p* > 0.05). Similarly, no significant between-intervention differences were observed in HRV indices at 30, 60, 90, or 120 min following ingestion (all *p* > 0.05) (Table 4).

Within-group analyses revealed time-dependent changes in specific HRV parameters. In the control jelly intervention, HF power (normalized units) was higher at 30 min compared with baseline (*p* = 0.001). No significant changes were observed in SDNN, RMSSD, LF power, HF power (absolute), or the LF/HF ratio over time (all *p* > 0.05).

In the *Hom Pathum* rice jelly intervention, SDNN at 60, 90, and 120 min was significantly higher than at baseline (*p* = 0.003, *p* < 0.001, and *p* = 0.001, respectively). Additionally, SDNN at 30 min was lower than at 90 min (*p* = 0.032). HF power increased at 30 min compared with baseline (*p* = 0.018), whereas RMSSD, LF power, and the LF/HF ratio remained unchanged (all *p* > 0.05).

In the *Tubtim Chumphae* rice jelly intervention, LF power at 60 min was lower than at baseline (*p* = 0.030). HF power (normalized units) increased at 30, 60, and 90 min compared with baseline (*p* = 0.020, *p* = 0.003, and *p* = 0.015, respectively), while the LF/HF ratio decreased at 30 and 60 min (*p* = 0.032 and *p* = 0.007, respectively). No significant changes were observed in SDNN, RMSSD, LF power (other time points), or HF power (absolute) (all *p* > 0.05).

#### 3.1.4. Cardiovascular Dynamic Response

Baseline cardiovascular parameters did not differ among interventions (all *p* > 0.05). Likewise, no significant between-intervention differences were observed in BP indices at 30, 60, 90, or 120 min following ingestion (all *p* > 0.05) (Table 5).

Within-group analyses showed no significant changes in SBP, DBP, PP, or MAP over time in any intervention (all *p* > 0.05). In the control jelly intervention, SBP, DBP, PP, and MAP remained unchanged (all *p* > 0.05). HR was lower at 30 and 90 min compared with baseline (*p* = 0.001 and *p* = 0.040, respectively), and RPP was reduced at 30 min (*p* = 0.011).

Similarly, in the *Hom Pathum* rice jelly intervention, HR and RPP at 30, 60, 90, and 120 min were significantly lower than at baseline (all *p* < 0.01).

In the *Tubtim Chumphae* rice jelly intervention, SBP, DBP, PP, and MAP also showed no significant changes over time (all *p* > 0.05). HR decreased at 30, 60, and 90 min compared with baseline (*p* < 0.001, *p* < 0.001, and *p* = 0.011, respectively), and was lower at 30 min than at 120 min (*p* = 0.047). RPP was reduced at 30, 60, and 90 min compared with baseline (*p* < 0.001, *p* < 0.001, and *p* = 0.025, respectively), with lower values at 30 min than at 120 min (*p* = 0.011).

### 3.2. Experiment 2

Similarly to Experiment 1, 25 participants were enrolled, and all (100%) completed the study. Therefore, data from all participants were included in the final analysis. However, the number of male participants (*n* = 6; 24%) was significantly lower than that of female participants (*n* = 19; 76%) (*p* = 0.009).

#### 3.2.1. Baseline Physical and Physiological Characteristics of Participants in Experiment 2

The baseline physical and physiological characteristics of the participants are presented in Table 6. Physical characteristics included age, height, body weight, BMI, body fat percentage, fat-free mass, muscle mass, protein mass, mineral mass, total body water, waist–hip ratio, visceral fat level, basal metabolic rate, and fitness score. Physiological characteristics included HR, SBP, DBP, resting VO_2_, and VO_2_peak.

#### 3.2.2. Endurance Performance

**Figure 5:**
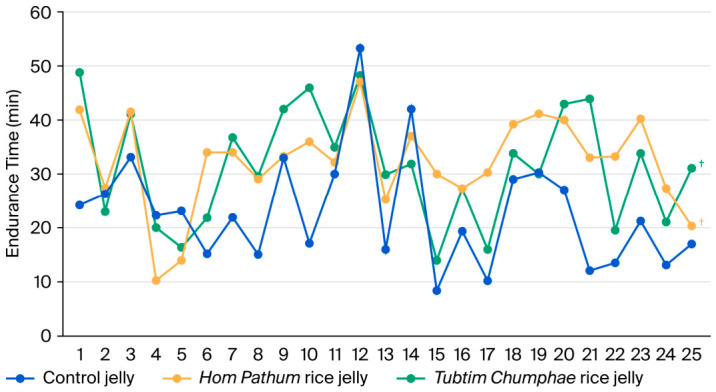
Endurance time to exhaustion during treadmill exercise performed at 60% VO_2_peak following ingestion of control jelly, *Hom Pathum* rice jelly, or *Tubtim Chumphae* rice jelly. Each data point represents an individual participant (*n* = 25). ^†^ *p* < 0.05 vs. control jelly.

Endurance time was significantly higher following *Hom Pathum* rice jelly (*p* = 0.004) and *Tubtim Chumphae* rice jelly (*p* = 0.001) compared with the control jelly. No significant difference was observed between the *Hom Pathum* and *Tubtim Chumphae* rice jellies (*p* = 0.772) (Figure 5; Table 7).

No significant differences were observed among interventions for HR, RPE, RPD, speed, or incline (all *p* > 0.05).

#### 3.2.3. Oxidative Stress Response to Endurance Exercise

**Figure 6:**
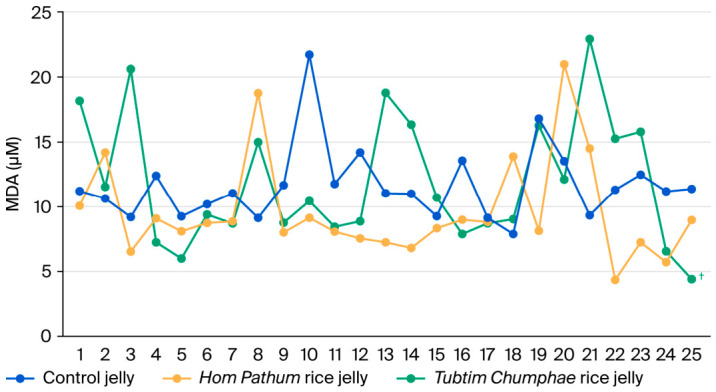
Post-exercise plasma malondialdehyde (MDA) concentrations following ingestion of control jelly, *Hom Pathum* rice jelly, or *Tubtim Chumphae* rice jelly. Each data point represents an individual participant (*n* = 25). MDA was measured immediately after the endurance test as an indicator of lipid peroxidation and oxidative stress. ^†^ *p* < 0.05 vs. control jelly.

MDA levels were significantly lower following *Tubtim Chumphae* rice jelly compared with the control jelly (*p* = 0.049). *Hom Pathum* rice jelly also showed a trend toward lower MDA levels compared with the control, although this did not reach statistical significance (*p* = 0.087) (Figure 6; Table 7). No significant difference was observed between the *Hom Pathum* and *Tubtim Chumphae* rice jellies (*p* = 0.089).

### 3.3. Chemical Constituents of Hom Pathum and Tubtim Chumphae Rice Grains and Rice Jellies

GC–MS analysis identified 12 and 16 compounds in *Hom Pathum* and *Tubtim Chumphae* rice grains, respectively. In *Hom Pathum* rice grains, propanoic acid, 3,3′-thiobis-, didodecyl ester (20.86% of the total peak area) and oleic acid (20.35%) were the predominant compounds, followed by hexadecanoic acid (16.75%), linoleic acid (16.00%), and dodecyl acrylate (12.59%). In contrast, *Tubtim Chumphae* rice grains were characterized by higher proportions of oleic acid (23.29%), linoleic acid (23.19%), and hexadecanoic acid (20.50%), with propanoic acid, 3,3′-thiobis-, didodecyl ester comprising 13.69% of the total peak area (Table 8).

Following jelly preparation, the chemical profiles of both rice varieties exhibited a similar pattern. Propanoic acid, 3,3′-thiobis-, didodecyl ester became the predominant constituent in *Hom Pathum* and *Tubtim Chumphae* rice jellies, accounting for 34.05% and 41.48% of the total peak area, respectively. Dodecyl acrylate, β-D-glucopyranose, 1,6-anhydro-, and 1-dodecanol were also detected as major constituents in both jelly formulations (Table 8).

## 4. Discussion

In this study, the acute effects of two Thai rice varieties, *Hom Pathum* and *Tubtim Chumphae*, on glycemic response, cardiovascular regulation, endurance performance, and oxidative stress in healthy adults were investigated. The principal findings were that both rice jellies attenuated postprandial BG responses and improved endurance performance compared with the glucose-based control, whereas *Tubtim Chumphae* rice additionally demonstrated potential to attenuate exercise-induced oxidative stress. These effects occurred without significant between-intervention differences in insulin concentrations, HOMA-derived indices, cardiac autonomic function, or BP, suggesting that physiological responses may involve mechanisms beyond acute insulin-mediated regulation.

Although these findings indicate potentially favorable acute physiological effects of *Hom Pathum* and *Tubtim Chumphae* rice jellies, they should be interpreted cautiously. In this study, short-term responses were evaluated in a relatively small cohort of healthy adults, but no long-term clinical outcomes or disease-related endpoints were assessed. Furthermore, detailed mechanistic characterization, including physicochemical and phytochemical determinants potentially underlying the observed responses, was beyond the scope of the present investigation. Accordingly, the present findings should be regarded as preliminary evidence supporting further investigation rather than definitive confirmation of functional or clinical efficacy.

The attenuation of postprandial BG concentrations and reduction in BG AUC following both rice interventions provide important insight into the potential metabolic relevance of Thai rice varieties. Despite their comparable caloric content and broadly similar sugar composition, both rice jellies elicited a more favorable glycemic profile than the glucose-based control [18]. These findings suggest that postprandial physiological responses depend not only on carbohydrate quantity but also on carbohydrate quality and food matrix characteristics [27]. Such effects may reflect differences in starch structure and physicochemical properties that influence digestion and glucose absorption kinetics [28], together with the presence of rice-derived phytochemicals, including phenolic and flavonoid compounds [29].

Notably, *Tubtim Chumphae* rice produced greater glycemic attenuation at selected postprandial time points. This observation may be partially associated with the phytochemical characteristics of pigmented rice varieties, particularly their anthocyanin content [30]. Anthocyanins have been reported to inhibit carbohydrate-digesting enzymes and modulate glucose transport pathways [31], thereby contributing to reduced postprandial glycemic excursions [32]. However, these proposed mechanisms should be interpreted cautiously. Although GC–MS analysis identified multiple chemical constituents in both rice grains and final jelly preparations, this study did not directly quantify total polyphenol or anthocyanin contents, evaluate enzyme inhibition, or assess phytochemical degradation during processing. Consequently, the observed glycemic effects cannot be attributed specifically to anthocyanins or polyphenols alone.

The lower postprandial glycemic responses observed following *Hom Pathum* and *Tubtim Chumphae* rice ingestion likely reflect the combined influence of rice variety, starch characteristics, processing conditions, and food matrix properties. Thermal processing represents an additional factor warranting consideration. *Hom Pathum* rice required boiling for 15 min, whereas *Tubtim Chumphae* rice required 30 min to achieve adequate cooking and rice milk extraction, reflecting inherent varietal differences in grain characteristics. Thermal processing influences starch gelatinization, granule disruption, food matrix organization, and the stability of heat-sensitive phytochemicals, including anthocyanins and phenolic compounds [33]. Such processing-induced modifications may subsequently alter starch digestibility and postprandial glycemic responses [27,28]. Therefore, the greater glycemic attenuation observed following *Tubtim Chumphae* rice consumption may reflect not only intrinsic compositional differences but also physicochemical changes induced during preparation.

Importantly, the present findings should also be interpreted within the context of comparator selection and food matrix characteristics. The glucose-based control jelly was designed to match caloric content and sugar composition while excluding rice-derived constituents, thereby permitting evaluation of physiological responses beyond carbohydrate intake alone. In addition, *Hom Pathum* rice jelly was intentionally included as a commonly consumed non-pigmented Thai rice comparator to provide a practical reference condition against which *Tubtim Chumphae* rice could be evaluated. Although this design enabled the assessment of both rice-derived and pigmentation-related characteristics, *Hom Pathum* rice also contains bioactive compounds and therefore does not represent an inert or low-bioactive comparator. Consequently, differences between the rice jellies should be interpreted as reflecting relative varietal and compositional differences rather than effects attributable exclusively to pigmented rice constituents.

Beyond compositional factors, physicochemical and rheological properties of the jellies may also have influenced the observed metabolic responses. Although all interventions were prepared using the same carrageenan-based formulation and standardized procedures, inherent differences in rice composition may have affected gel structure, texture, and disintegration behavior. Such characteristics influence gastric breakdown, nutrient release kinetics, and subsequent postprandial glycemic and hormonal responses. Because gel strength, viscosity, and other rheological parameters were not evaluated, their contribution cannot be excluded. Accordingly, the present findings should be interpreted as reflecting the combined effects of rice composition, food matrix characteristics, and processing conditions rather than the isolated actions of specific bioactive compounds alone.

Despite the observed differences in BG responses, no significant between-intervention effects were identified for insulin concentrations or HOMA-derived indices. These findings suggest that improved glycemic responses were more likely mediated through reduced glucose absorption [34] or altered carbohydrate handling [35] rather than enhanced insulin secretion or sensitivity during the acute postprandial period. Such observations are consistent with previous studies demonstrating that polyphenol-rich foods may modulate postprandial glycemia independently of measurable changes in insulin dynamics [36,37,38,39,40].

Similarly, no significant between-intervention differences were observed in HRV or BP indices, indicating that acute rice jelly consumption did not markedly influence autonomic cardiovascular regulation in healthy individuals. Although modest within-condition increases in parasympathetic activity were observed, particularly following *Tubtim Chumphae* rice ingestion, these changes were insufficient to produce statistically significant between-condition effects. Previous investigations have reported improvements in autonomic cardiovascular regulation following consumption of polyphenol-rich foods through the modulation of parasympathetic and sympathetic activity [41,42,43]. However, discrepancies between studies may reflect differences in participant characteristics, intervention duration, and the type and dose of bioactive compounds administered.

Despite the absence of significant autonomic or cardiovascular effects, both rice interventions significantly improved endurance performance. Time to exhaustion increased following both *Hom Pathum* and *Tubtim Chumphae* rice consumption compared with under the control condition (+8.46 min and +9.25 min, respectively), despite comparable HR, perceived dyspnea, leg fatigue, and exercise workload across interventions. This pattern suggests that the observed performance enhancement was unlikely to be mediated primarily through cardiovascular or perceptual mechanisms and may instead reflect alterations in metabolic efficiency or substrate availability [41].

One plausible explanation is that attenuation of postprandial glycemic fluctuations following rice jelly ingestion provided a more stable and sustained energy supply during exercise [44], thereby delaying fatigue onset and prolonging endurance capacity. Differences in starch digestibility and glucose release kinetics may have contributed to this effect, particularly given the more favorable postprandial glycemic profiles observed following both rice interventions relative to the glucose-based control. In addition, rice-derived phytochemicals may support cellular energy metabolism [45] or attenuate exercise-associated oxidative perturbations [46], although these mechanisms were not directly evaluated in this study. Consistent with the current findings, previous studies have reported that polyphenol-rich foods may enhance physical performance, including endurance capacity [47,48,49]. Nevertheless, because substrate metabolism and energy utilization were not assessed, the precise physiological mechanisms underlying the observed performance benefits remain uncertain.

The reduction in post-exercise MDA concentration following *Tubtim Chumphae* rice ingestion provides further insight into the potential functional relevance of pigmented rice consumption. MDA is a commonly used marker of lipid peroxidation and oxidative stress that typically increases following endurance exercise [50]. The attenuation observed in this study suggests that *Tubtim Chumphae* rice may exert acute antioxidant effects and thereby mitigate exercise-induced oxidative stress. Such effects may be associated with the greater abundance of anthocyanins and related polyphenolic compounds commonly present in pigmented rice varieties [51]. Previous investigations have similarly demonstrated that polyphenol-rich foods, particularly those containing pigmented phytochemicals such as anthocyanins, may attenuate exercise-induced oxidative stress and support redox balance [52,53,54].

However, interpretation of the antioxidant findings requires caution. Although *Hom Pathum* rice demonstrated a comparable directional trend, the reduction in MDA did not achieve statistical significance. This difference may reflect variation in phytochemical composition between rice varieties [55], inter-individual variability in oxidative stress responses [56], or limited statistical power for oxidative stress outcomes. Moreover, mechanistic biomarkers related to antioxidant capacity, inflammatory regulation, muscle damage, and substrate metabolism were not evaluated. Consequently, the biological pathways underlying the observed reduction in oxidative stress cannot be fully elucidated from the present findings alone.

Several strengths of the present study should be acknowledged. Its randomized crossover design minimized inter-individual variability and strengthened internal validity, while the inclusion of both metabolic and exercise-related outcomes permitted broader evaluation of the acute physiological effects of rice-based interventions. Furthermore, the incorporation of both a glucose-based control and a commonly consumed non-pigmented Thai rice comparator enabled the assessment of physiological responses attributable to rice consumption and facilitated contextual interpretation of pigmentation-related characteristics. Nevertheless, several limitations warrant consideration. First, Experiment 2 did not include BG, insulin, or oxidative stress assessments immediately prior to endurance exercise. Consequently, within-condition changes and exercise-related alterations in glycemic regulation and oxidative stress could not be comprehensively characterized. Second, although the proximate composition, caloric content, and major chemical constituents of the rice jellies were evaluated, several potentially relevant compositional and physicochemical properties were not determined. Specifically, resistant starch content, the amylose-to-amylopectin ratio, dietary fiber content, glycemic index characterization, rheological properties, and the comprehensive polyphenol and anthocyanin profiles of the final jelly products were not assessed. These characteristics may substantially influence gel structure, gastric disintegration, starch digestibility, glucose release kinetics, and antioxidant activity and may have therefore contributed to the physiological responses observed in this study. Third, targeted mechanistic assessments were not performed. Biomarkers related to antioxidant capacity, inflammatory cytokines, muscle damage, and substrate metabolism were not measured, thereby limiting mechanistic interpretation of the observed metabolic and endurance-related responses. Fourth, this study examined only acute physiological responses, and therefore, the long-term effects and clinical relevance of habitual rice jelly consumption remain unknown. Additionally, participants were young and healthy, which may limit generalizability to populations with impaired glucose regulation or chronic metabolic disease. Finally, the sex distribution of participants in Experiment 2 was also uneven, with female participants comprising 76% of the cohort. Given recognized sex-related differences in metabolic regulation, substrate utilization, hormonal responses, and exercise performance, this imbalance may limit generalizability across sexes. Furthermore, the relatively small number of male participants precluded adequately powered sex-stratified analyses. Accordingly, the present findings should be interpreted with appropriate caution.

Future research incorporating targeted phytochemical characterization and mechanistic investigation is warranted to clarify the pathways underlying the observed physiological responses. In particular, direct assessment of phytochemical stability, starch gelatinization, and digestibility under standardized processing conditions is needed to distinguish the relative contributions of rice variety and thermal processing to glycemic responses. More comprehensive characterization of final jelly products, including resistant starch content, the amylose-to-amylopectin ratio, rheological properties, and detailed polyphenol and anthocyanin profiling, would strengthen mechanistic interpretation and improve reproducibility of rice-based nutritional interventions. Future studies should additionally incorporate biomarkers related to oxidative stress, inflammation, antioxidant capacity, muscle damage, and substrate metabolism to better elucidate biological mechanisms. Longer-term investigations are also required to determine whether the acute physiological responses observed in this study translate into clinically meaningful outcomes. Such research should include more diverse and metabolically relevant populations, including individuals with insulin resistance or type 2 diabetes, and incorporate adequately powered sex-specific analyses to clarify potential sex-dependent responses to rice-based interventions and endurance exercise.

## 5. Conclusions

The present findings suggest that *Hom Pathum* and *Tubtim Chumphae* rice jellies may differentially affect acute glycemic responses and endurance performance in healthy adults, with *Tubtim Chumphae* rice additionally demonstrating potential to attenuate exercise-induced oxidative stress. However, these observations reflect short-term physiological responses under controlled experimental conditions and should not be interpreted as evidence of established long-term clinical benefit, health promotion, or disease-preventive effects. Further studies involving larger and more diverse populations, longer intervention durations, and detailed mechanistic characterization are warranted to confirm and better understand these observed physiological responses.

## Figures and Tables

**Table 1:** Nutritional composition of control jelly, *Hom Pathum* rice jelly, and *Tubtim Chumphae* rice jelly used in the intervention studies.

Composition	Control Jelly	*Hom Pathum* Rice Jelly	*Tubtim Chumphae* Rice Jelly
Total carbohydrate (g/100 g)	10.60	9.97	9.62
Total protein (g/100 g)	ND	0.62	0.87
Total fat (g/100 g)	ND	0.02	0.03
Total sugars (g/100 g)	10.00	7.81	7.96
Moisture (g/100 g)	98.54	96.22	95.55
Ash (g/100 g)	ND	0.03	0.15
Sodium (mg/100 g)	2.30	2.82	3.40
Potassium (mg/100 g)	ND	16.01	77.20
Energy (kcal/100 g)	40.00	39.90	39.60

Nutritional composition was analyzed by the National Food Institute, Thailand. Ash, moisture, and total fat contents were determined using AOAC methods. Total protein, total sugars, sodium, and potassium were determined using validated in-house AOAC-based methods. Total carbohydrate and energy contents were calculated. Values are expressed as g/100 g, mg/100 g, or kcal/100 g, as indicated. ND, not detected.

**Table 2:** Baseline demographic, anthropometric, and cardiovascular characteristics of participants in Experiment 1.

Characteristic	Mean ± SD	Minimum	Maximum
Sex (*n*, %)			
Male	12 (48)	-	-
Female	13 (52)	-	-
Age (years)	20.76 ± 1.20	18	24
Body weight (kg)	59.33 ± 9.72	41.1	78.7
Body mass index (kg/m^2^)	21.00 ± 2.69	16.7	27
Body fat (%)	23.38 ± 8.34	10.2	44.8
Subcutaneous fat (%)	20.00 ± 7.31	7.1	36.8
Muscle (%)	30.72 ± 4.25	24	38.6
Visceral fat level	3.92 ± 2.81	0.5	10.5
Basal metabolic rate (kcal)	1359.12 ± 218.57	1009	1827
HR (/min)	76.54 ± 10.47	52	101
SBP (mmHg)	105.69 ± 10.67	78	138
DBP (mmHg)	65.78 ± 6.31	48	80

Data are presented as mean ± standard deviation (SD), frequencies, or percentages as appropriate (*n* = 25). Body composition and physiological variables were assessed prior to intervention. DBP, diastolic blood pressure; HR, heart rate; SBP, systolic blood pressure.

**Table 3:** Blood glucose concentrations, glucose area under the curve (AUC), insulin concentrations, and HOMA indices before and after ingestion of control jelly, *Hom Pathum* rice jelly, and *Tubtim Chumphae* rice jelly.

	Control Jelly	*Hom Pathum* Rice Jelly	*Tubtim Chumphae* Rice Jelly	Two-Way ANOVA (*p* Value)		
				Intervention	Time	Interaction
Glucose (mg/dL)						
0 min	97.24 ± 12.19	93.75 ± 7.47	88.76 ± 9.19	<0.05	<0.05	<0.05
30 min	120.33 ± 13.03 *	111.24 ± 13.81 *^,^^†,‡^	99.68 ± 11.73 *^,†^			
60 min	111.54 ± 13.19 *	96.96 ± 10.88 ^†^	95.24 ± 10.22 ^†^			
90 min	99.29 ± 8.99	93.64 ± 12.98 ^†^	93.32 ± 7.60 ^†^			
120 min	94.29 ± 7.39 *	92.96 ± 8.02	87.68 ± 10.79 ^†^			
AUC of glucose (mg/dL·h)						
0–0.5 h	53.52 ± 4.39	50.00 ± 5.22 ^†^	49.23 ± 5.68 ^†^	<0.001	<0.001	<0.001
0–1.0 h	111.49 ± 9.56	102.05 ± 10.39 ^†^	97.96 ± 10.81 ^†^			
0–1.5 h	164.20 ± 13.06	149.70 ± 15.06 ^†^	145.10 ± 14.91 ^†^			
0–2.0 h	212.59 ± 15.03	195.03 ± 19.59 ^†^	191.67 ± 18.15 ^†^			
Insulin (µU/mL)						
0 min	10.53 ± 24.51	10.50 ± 9.09	9.55 ± 9.17	>0.05	0.002	0.069
120 min	8.58 ± 9.34 *	7.59 ± 6.71 *	6.27 ± 3.71 *			
HOMA-IR						
0 min	2.52 ± 6.00	2.40 ± 2.00	2.21 ± 2.46	>0.05	0.003	0.058
120 min	1.93 ± 1.90	1.76 ± 1.78 *	1.46 ± 0.85 *			
HOMA-B						
0 min	4.24 ± 2.96	5.29 ± 5.54	6.65 ± 9.97	>0.05	0.007	0.933
120 min	7.97 ± 10.12	10.30 ± 18.91	12.08 ± 19.97			
HOMA-%S						
0 min	63.93 ± 50.98	66.15 ± 42.16	84.44 ± 77.92	>0.05	0.044	0.334
120 min	82.63 ± 67.49	111.62 ± 89.69	153.24 ± 286.14 *			
QUICKI						
0 min	3.19 ± 0.54	3.21 ± 0.46	4.08 ± 4.60	>0.05	0.532	0.392
120 min	3.54 ± 1.53	3.69 ± 1.23	4.22 ± 4.74			

Data are presented as mean ± SD (*n* = 25). Time-dependent responses and intervention effects were analyzed using repeated-measures two-way ANOVA/ANCOVA as indicated. AUC was calculated for postprandial blood glucose responses over the 2 h observation period. HOMA-B, homeostatic model assessment of beta-cell function; HOMA-IR, homeostatic model assessment of insulin resistance; HOMA-%S, homeostatic model assessment of insulin sensitivity; QUICKI, quantitative insulin sensitivity check index. * *p* < 0.05 vs. baseline; ^†^ *p* < 0.05 vs. control jelly; ^‡^ *p* < 0.05 vs. *Tubtim Chumphae* rice jelly.

**Table 4:** Heart rate variability (HRV) responses following ingestion of control jelly, *Hom Pathum* rice jelly, and *Tubtim Chumphae* rice jelly during the 120 min postprandial period.

	Control Jelly	*Hom Pathum* Rice Jelly	*Tubtim Chumphae* Rice Jelly	Two-Way ANOVA (*p* Value)		
				Intervention	Time	Interaction
SDNN (ms)						
0 min	86.01 ± 53.75	68.25 ± 28.78	79.32 ± 32.12	>0.05	>0.05	0.046
30 min	83.56 ± 39.46	76.26 ± 28.62	80.99 ± 30.66			
60 min	87.54 ± 56.80	85.18 ± 30.14 *	79.80 ± 31.07			
90 min	86.48 ± 32.98	87.62 ± 27.62 *	88.67 ± 36.73			
120 min	85.62 ± 29.55	85.69 ± 26.14 *	95.37 ± 37.90			
RMSSD (ms)						
0 min	64.07 ± 32.56	82.93 ± 57.74	79.24 ± 42.86	>0.05	>0.05	0.233
30 min	67.99 ± 33.34	83.97 ± 63.09	78.07 ± 42.62			
60 min	71.25 ± 31.37	83.94 ± 84.97	81.00 ± 44.81			
90 min	77.52 ± 34.44	74.22 ± 41.21	89.95 ± 50.07			
120 min	68.55 ± 32.89	71.53 ± 34.01	83.55 ± 43.44			
LF power × 10^−2^ (ms^2^)						
0 min	16.19 ± 15.75	22.90 ± 28.09	19.10 ± 22.15	>0.05	>0.05	0.126
30 min	16.14 ± 18.28	15.45 ± 13.14	18.11 ± 19.57			
60 min	15.67 ± 13.45	21.57 ± 33.06	14.31 ± 18.66 *			
90 min	20.86 ± 18.07	16.16 ± 17.21	19.72 ± 24.13			
120 min	17.22 ± 12.64	15.53 ± 12.77	20.50 ± 19.85			
LF (n.u.)						
0 min	44.25 ± 16.70	45.23 ± 14.13	42.53 ± 13.74	<0.05	<0.05	0.337
30 min	40.90 ± 16.42	37.91 ± 14.31	36.10 ± 13.45			
60 min	40.94 ± 16.10	40.82 ± 19.65	32.20 ± 15.25			
90 min	43.46 ± 16.55	45.24 ± 20.72	36.32 ± 14.94			
120 min	48.07 ± 15.46	44.20 ± 20.68	43.34 ± 17.39			
HF power × 10^−2^ (ms^2^)						
0 min	15.77 ± 16.82	20.31 ± 17.96	22.54 ± 23.14	>0.05	>0.05	0.276
30 min	23.28 ± 17.74	33.40 ± 75.07 *	28.85 ± 28.04			
60 min	18.10 ± 15.22	30.76 ± 49.64	25.46 ± 23.42			
90 min	19.52 ± 16.92	30.25 ± 40.64	32.77 ± 31.18			
120 min	18.59 ± 15.55	22.78 ± 24.69	26.37 ± 23.94			
HF (n.u.)						
0 min	42.71 ± 17.55	40.21 ± 16.34	46.89 ± 14.80	<0.05	<0.05	0.125
30 min	50.81 ± 17.74 *	53.52 ± 17.82	55.10 ± 14.77 *			
60 min	48.94 ± 17.73	50.54 ± 17.55	59.44 ± 14.45 *			
90 min	49.46 ± 18.07	50.47 ± 19.88	56.62 ± 14.18 *			
120 min	44.00 ± 16.63	49.58 ± 23.48	48.43 ± 16.53			
LF/HF ratio						
0 min	1.52 ± 1.47	1.59 ± 1.46	1.07 ± 0.62	>0.05	<0.05	0.990
30 min	1.16 ± 1.12	1.03 ± 1.14	0.74 ± 0.42 *			
60 min	1.01 ± 0.67	1.43 ± 2.50	0.65 ± 0.39 *			
90 min	1.17 ± 0.90	1.40 ± 1.72	0.76 ± 0.56			
120 min	1.55 ± 1.56	1.73 ± 2.21	1.12 ± 0.77			

Data are presented as mean ± SD (*n* = 25). HRV parameters were assessed at baseline and throughout the postprandial period to evaluate cardiac autonomic nervous system activity. Statistical analyses were performed using repeated-measures ANOVA or ANCOVA, as appropriate, to assess time and intervention effects. HF, high-frequency power; LF, low-frequency power; LF/HF, ratio of low- to high-frequency power; RMSSD, root mean square of successive R–R interval differences; SDNN, standard deviation of normal-to-normal (NN) intervals. * *p* < 0.05 vs. baseline within the same intervention condition.

**Table 5:** Blood pressure and cardiovascular responses following ingestion of control jelly, *Hom Pathum* rice jelly, and *Tubtim Chumphae* rice jelly during the postprandial period.

	Control Jelly	*Hom Pathum* Rice Jelly	*Tubtim Chumphae* Rice Jelly	Two-Way ANOVA (*p* Value)		
				Intervention	Time	Interaction
HR (/min)						
0 min	76.13 ± 10.96	77.72 ± 9.68	75.76 ± 11.07	>0.05	<0.05	0.170
30 min	66.00 ± 12.66 *	65.76 ± 9.28 *	63.24 ± 10.27 *			
60 min	69.83 ± 13.49	68.00 ± 10.68 *	64.16 ± 9.73 *			
90 min	69.00 ± 12.86 *	69.68 ± 9.09 *	67.32 ± 11.96 *			
120 min	71.25 ± 9.56	66.00 ± 9.78 *	69.84 ± 8.57			
SBP (mmHg)						
0 min	106.04 ± 10.05	105.08 ± 9.97	105.92 ± 12.22	>0.05	>0.05	0.985
30 min	104.40 ± 9.54	104.79 ± 7.55	103.60 ± 9.39			
60 min	104.72 ± 10.92	103.71 ± 7.20	102.32 ± 9.18			
90 min	105.76 ± 7.83	104.79 ± 7.84	104.92 ± 11.05			
120 min	105.80 ± 9.37	104.42 ± 10.31	105.32 ± 19.54			
DBP (mmHg)						
0 min	65.84 ± 7.12	65.00 ± 4.35	66.48 ± 7.14	>0.05	<0.05	0.536
30 min	67.36 ± 5.24	65.46 ± 4.81	63.92 ± 5.31			
60 min	67.80 ± 7.09	66.29 ± 4.02	67.20 ± 6.56			
90 min	68.44 ± 6.08	67.54 ± 4.36	66.44 ± 5.24			
120 min	67.16 ± 6.07	65.17 ± 8.56	66.56 ± 7.88			
PP (mmHg)						
0 min	40.20 ± 9.25	40.08 ± 9.12	39.44 ± 11.27	>0.05	<0.05	0.898
30 min	37.04 ± 8.75	39.33 ± 7.83	39.68 ± 8.99			
60 min	36.92 ± 7.51	37.42 ± 7.08	35.12 ± 10.53			
90 min	37.32 ± 5.88	37.25 ± 8.35	38.48 ± 8.85			
120 min	38.64 ± 9.78	39.25 ± 12.28	38.76 ± 18.02			
MAP (mmHg)						
0 min	79.24 ± 6.96	78.36 ± 5.23	79.63 ± 7.45	>0.05	<0.05	0.777
30 min	79.71 ± 5.62	78.57 ± 4.56	77.15 ± 5.50			
60 min	80.11 ± 7.79	78.76 ± 4.11	78.91 ± 5.67			
90 min	80.88 ± 6.12	79.96 ± 4.20	79.27 ± 6.45			
120 min	80.04 ± 5.71	78.25 ± 7.13	79.48 ± 9.82			
RPP × 10^−2^ (mmHg·beats/min)						
0 min	79.85 ± 12.67	82.38 ± 12.73	80.05 ± 14.38	>0.05	<0.05	0.289
30 min	69.33 ± 15.12 *	68.81 ± 12.63 *	65.35 ± 11.89 *			
60 min	72.40 ± 14.53	71.22 ± 13.28 *	65.70 ± 11.93 *			
90 min	72.49 ± 15.31	73.79 ± 12.30 *	70.88 ± 16.09 *			
120 min	74.31 ± 11.57	69.93 ± 12.33 *	73.75 ± 18.41			

Data are presented as mean ± SD (*n* = 25). Cardiovascular variables were assessed at baseline and throughout the 120 min postprandial period following ingestion of the experimental jellies. Statistical analyses were performed using repeated-measures ANOVA or ANCOVA, as appropriate, to assess temporal and between-intervention effects. BP, blood pressure; SBP, systolic blood pressure; DBP, diastolic blood pressure; HR, heart rate. * *p* < 0.05 vs. baseline within the same intervention condition.

**Table 6:** Baseline demographic, anthropometric, and physiological characteristics of participants in Experiment 2.

Characteristic	Mean ± SD	Minimum	Maximum
Sex (*n*, %)			
Male	6 (24)	-	-
Female	19 (76)	-	-
Age (years)	20.88 ± 0.78	20	22
Height (cm)	163.64 ± 8.40	153	183
Body weight (kg)	56.15 ± 10.46	42.7	83
Body mass index (kg/m^2^)	20.84 ± 2.45	17.9	25.9
Body fat (%)	27.18 ± 8.16	8.2	40.4
Fat-free mass (kg)	15.26 ± 5.47	4.4	25.5
Muscle mass (kg)	22.20 ± 5.46	15.6	34.5
Protein mass (kg)	8.02 ± 1.80	5.8	12.2
Mineral mass (kg)	2.92 ± 0.57	2.15	4.23
Water mass (kg)	29.94 ± 6.64	21.7	44.7
Waist–hip ratio	0.83 ± 0.05	0.76	0.99
Visceral fat level	6.07 ± 2.94	1	12
Basal metabolic rate (kcal)	1253.16 ± 194.38	1011	1690
Fitness score	70.24 ± 3.98	62	78
HR (/min)	76.43 ± 13.31	53	101
SBP (mmHg)	115.56 ± 9.98	100	136
DBP (mmHg)	69.12 ± 8.34	49	94
Resting VO_2_ (L/min)	4.96 ± 0.84	3	6
VO_2_peak (L/min)	28.20 ± 5.36	20	40

Data are presented as mean ± SD, frequencies, or percentages, as appropriate (*n* = 25). Body composition, cardiovascular parameters, and aerobic fitness indicators were assessed prior to intervention. DBP, diastolic blood pressure; HR, heart rate; SBP, systolic blood pressure; VO_2_, oxygen consumption; VO_2_peak, peak oxygen consumption.

**Table 7:** Endurance performance, oxidative stress biomarker concentrations, and cardiorespiratory responses measured following ingestion of control jelly, *Hom Pathum* rice jelly, and *Tubtim Chumphae* rice jelly during treadmill exercise performed at matched speed and incline levels.

	Control Jelly	*Hom Pathum* Rice Jelly	*Tubtim Chumphae* Rice Jelly	One-Way ANOVA	
				*F* Value	*p* Value
Endurance time (min)	22.98 ± 10.40	31.41 ± 10.56	32.23 ± 8.65	6.682	<0.05
MDA (µM)	11.93 ± 4.95	10.63 ± 2.87	9.67 ± 3.91	2.366	>0.05
HR (bpm)	153.20 ± 15.32	152.20 ± 16.72	152.32 ± 20.32	0.024	>0.05
RPE score	15.28 ± 2.15	16.00 ± 2.20	16.12 ± 2.11	1.113	>0.05
RPD score	5.28 ± 2.15	6.00 ± 2.20	6.12 ± 2.11	1.113	>0.05
Speed (km/h)	4.42 ± 0.64	4.37 ± 0.61	4.37 ± 0.61	0.067	>0.05
Inclination (%)	12.56 ± 0.92	12.48 ± 0.87	12.48 ± 0.87	0.068	>0.05

Data are presented as mean ± SD (*n* = 25). Endurance exercise was performed at 60% VO_2_peak until exhaustion. Blood samples for oxidative stress assessment were collected immediately after exercise. Between-intervention differences were evaluated using one-way ANOVA. HR, heart rate; MDA, malondialdehyde; RPD, rating of perceived dyspnea; RPE, rating of perceived exertion.

**Table 8:** Chemical constituents in *Hom Pathum* and *Tubtim Chumphae* rice grains and corresponding rice jelly products identified via gas chromatography–mass spectrometry (GC–MS).

Constituent	*Hom Pathum* Rice (% Area Sum)		*Tubtim Chumphae* Rice (% Area Sum)	
	Grains	Jelly	Grains	Jelly
1-Dodecanol	4.19	10.65	2.79	10.13
Dodecyl acrylate	12.59	21.25	6.99	17.51
Tetradecanoic acid	0.64	0.72	0.57	0.70
Hexadecanoic acid	16.75	4.05	20.50	4.05
Hexadecanoic acid, ethyl ester	ND	ND	0.46	ND
Propanoic acid, 3-mercapto-, dodecyl ester	2.89	4.86	1.63	4.06
Linoleic acid	16.00	0.86	23.19	0.72
Oleic Acid	20.35	2.49	23.29	3.28
Octadecanoic acid	1.18	1.29	1.62	1.26
Butyl 9,12-octadecadienoate	ND	ND	0.19	ND
Acetic acid, (butylthio)-, methyl ester	ND	ND	0.44	ND
Stigmast-5-en-3-ol, oleate	0.83	ND	0.96	ND
Ergost-5-en-3-yl acetate	0.89	ND	0.62	ND
Cholesta-6,22,24-triene, 4,4-dimethyl-	ND	ND	0.28	ND
Stigmastan-3,5-diene	2.83	ND	2.78	ND
Propanoic acid, 3,3′-thiobis-, didodecyl ester	20.86	34.05	13.69	41.48
Glycerin	ND	ND	ND	3.70
Isosorbide	ND	3.07	ND	1.53
β-D-Glucopyranose, 1,6-anhydro-	ND	16.72	ND	11.57

Relative abundance of identified compounds is expressed as percentage peak area (% area sum). Chemical analyses were performed by the Center for Scientific and Technological Equipment (CSTE), Suranaree University of Technology, Thailand. *Hom Pathum* and *Tubtim Chumphae* samples were analyzed both before (grains) and after jelly preparation (jelly). ND, not detected.

## Data Availability

The data are available upon request from the corresponding author. Restrictions apply to the availability of these data due to privacy and ethical considerations.

## Abbreviations

The following abbreviations are used in this manuscript:

ANCOVA	Analysis of covariance
ANOVA	Analysis of variance
AUC	Area under the curve
BG	Blood glucose
BMI	Body mass index
BP	Blood pressure
DBP	Diastolic blood pressure
EDTA	Ethylenediaminetetraacetic acid
HF	High frequency
HOMA	Homeostatic model assessment
HOMA-B	Homeostasis model assessment of beta-cell function
HOMA-IR	Homeostatic model assessment of insulin resistance
HOMA-%S	Homeostatic model assessment for insulin sensitivity
HR	Heart rate
HRV	Heart rate variability
LF	Low frequency
MAP	Mean arterial pressure
MDA	Malondialdehyde
PP	Pulse pressure
QUICKI	Quantitative insulin sensitivity check index
RMSSD	Root mean square of successive R–R interval differences
RPD	Rating of perceived dyspnea
RPE	Rating of perceived exertion
RPP	Rate–pressure product
SBP	Systolic blood pressure
SD	Standard deviation
SDNN	Standard deviation of normal-to-normal intervals
TBA	Thiobarbituric acid
TBAR	Thiobarbituric acid reactive substances
VO_2_peak	Peak oxygen consumption
